# FARP1 boosts CDC42 activity from integrin αvβ5 signaling and correlates with poor prognosis of advanced gastric cancer

**DOI:** 10.1038/s41389-020-0190-7

**Published:** 2020-02-06

**Authors:** Takuro Hirano, Yoshinari Shinsato, Kan Tanabe, Nayuta Higa, Muhammad Kamil, Kohichi Kawahara, Masatatsu Yamamoto, Kentaro Minami, Michiko Shimokawa, Takaaki Arigami, Shigehiro Yanagita, Daisuke Matushita, Yoshikazu Uenosono, Sumiya Ishigami, Yuko Kijima, Kosei Maemura, Ikumi Kitazono, Akihide Tanimoto, Tatsuhiko Furukawa, Shoji Natsugoe

**Affiliations:** 1 Department of Digestive Surgery, Breast and Thyroid Surgery, Kagoshima University Graduate School of Medical and Dental Sciences, Kagoshima, Japan; 2 Department of Molecular Oncology, Kagoshima University Graduate School of Medical and Dental Sciences, Kagoshima, Japan; 3 Onco-Biological Surgery, Kagoshima University Graduate School of Medical and Dental Sciences, Kagoshima, Japan; 4 Department of Neurosurgery, Kagoshima University Graduate School of Medical and Dental Sciences, Kagoshima, Japan; 5 Center for the Research of Advanced Diagnosis and Therapy of Cancer, Graduate School of Medical and Dental Sciences, Kagoshima University, Kagoshima, Japan; 6 Department of Pathology, Kagoshima University Graduate School of Medical and Dental Sciences, Kagoshima, Japan

**Keywords:** Gastric cancer, Targeted therapies, Target identification, Extracellular matrix, RHO signalling

## Abstract

Considering the poor prognosis of most advanced cancers, prevention of invasion and metastasis is essential for disease control. Ras homologous (Rho) guanine exchange factors (GEFs) and their signaling cascade could be potential therapeutic targets in advanced cancers. We conducted in silico analyses of The Cancer Genome Atlas expression data to identify candidate Rho-GEF genes showing aberrant expression in advanced gastric cancer and found *FERM, Rho/ArhGEF, and pleckstrin domain protein 1* (*FARP1*) expression is related to poor prognosis. Analyses in 91 clinical advanced gastric cancers of the relationship of prognosis and pathological factors with immunohistochemical expression of FARP1 indicated that high expression of FARP1 is significantly associated with lymphatic invasion, lymph metastasis, and poor prognosis of the patients (*P* = 0.025). In gastric cancer cells, FARP1 knockdown decreased cell motility, whereas FARP1 overexpression promoted cell motility and filopodium formation via CDC42 activation. FARP1 interacted with integrin β5, and a potent integrin αvβ5 inhibitor (SB273005) prevented cell motility in only high FARP1-expressing gastric cancer cells. These results suggest that the integrin αvβ5-FARP1-CDC42 axis plays a crucial role in gastric cancer cell migration and invasion. Thus, regulatory cascade upstream of Rho can be a specific and promising target of advanced cancer treatment.

## Introduction

Molecular targeted therapies have successfully improved prognoses of several patients with cancer; however, prognoses of most patients carrying advanced cancers are still poor. In fact, trastuzumab (a HER2-neutralizing antibody) and ramucirumab (an anti-VEGFR-2 antibody) have been introduced with or without combined treatment of cytotoxic agents that have improved the survival of patients with gastric cancer; however, the overall survival of patients with advanced gastric cancer remains discouraging^1^. Gastric cancer remains the second leading cause of cancer-related deaths worldwide^2,3^. The only curative treatment for advanced gastric cancer is surgery. The prognosis of patients with metastatic gastric cancer is poor, with median survival ranging from 4 to 12 months, depending on the medical treatments applied^4,5^. Therefore, better management of advanced cancers, including gastric cancer, particularly through the use of new targeted therapeutic agents, is urgently required.

Recent studies have revealed the aberrant expression of or genetic alterations in Ras homologous (Rho) guanine exchange factors (GEFs) in several human cancers^6–10^, which is consistent with their reported crucial role in the deregulated signaling of human cancer initiation and progression^11^. Rho family proteins comprises 20 members in humans as a major branch of the Ras superfamily of small GTPases that specifically regulate actin organization, cell motility, polarity, growth, survival, and gene transcription^1,12^. Rho family proteins act as binary switches that are highly regulated by Rho GEFs that induce the replacement of bound GDP by GTP. In human cancers, Rho GTPases are crucial for cancer cell migration, invasion, and metastasis^13^. Accordingly, mutations of Ras genes have been identified in over 30% of human cancers^14^; conversely, very few mutations in Rho GTPases have been detected.

FERM, Rho/ArhGEF, and pleckstrin domain protein 1 (FARP1) constitutes a Rho GEF protein that is composed of an ezrin-like domain, which is found in cytoskeleton-associated proteins of the band 4.1 superfamily, a Dbl homology (DH) domain, and two pleckstrin homology (PH) domains, which are conserved in Rho GEF family members^15^. Recently, it was reported that in dendrites, FARP1 binds SynCAM1 and integrates excitatory synapse development via Rac1 activation^16^, whereas in endothelial cells, it regulates the endothelial barrier via a signaling unit also comprising PAK7, a CDC42 effector, and the CDC42-GTPase-activating protein SYDE1^17^. However, the impact of FARP1 expression in cancer remains poorly understood.

In the present study, we examined correlation between FARP1 expression and the prognosis of patients with gastric cancer, and explored the potential role of the integrin αvβ5-FARP1-CDC42 axis in promoting cancer cell migration and invasion.

## Results

### Identification of candidate Rho GEF genes in gastric cancer

**Fig. 1:**
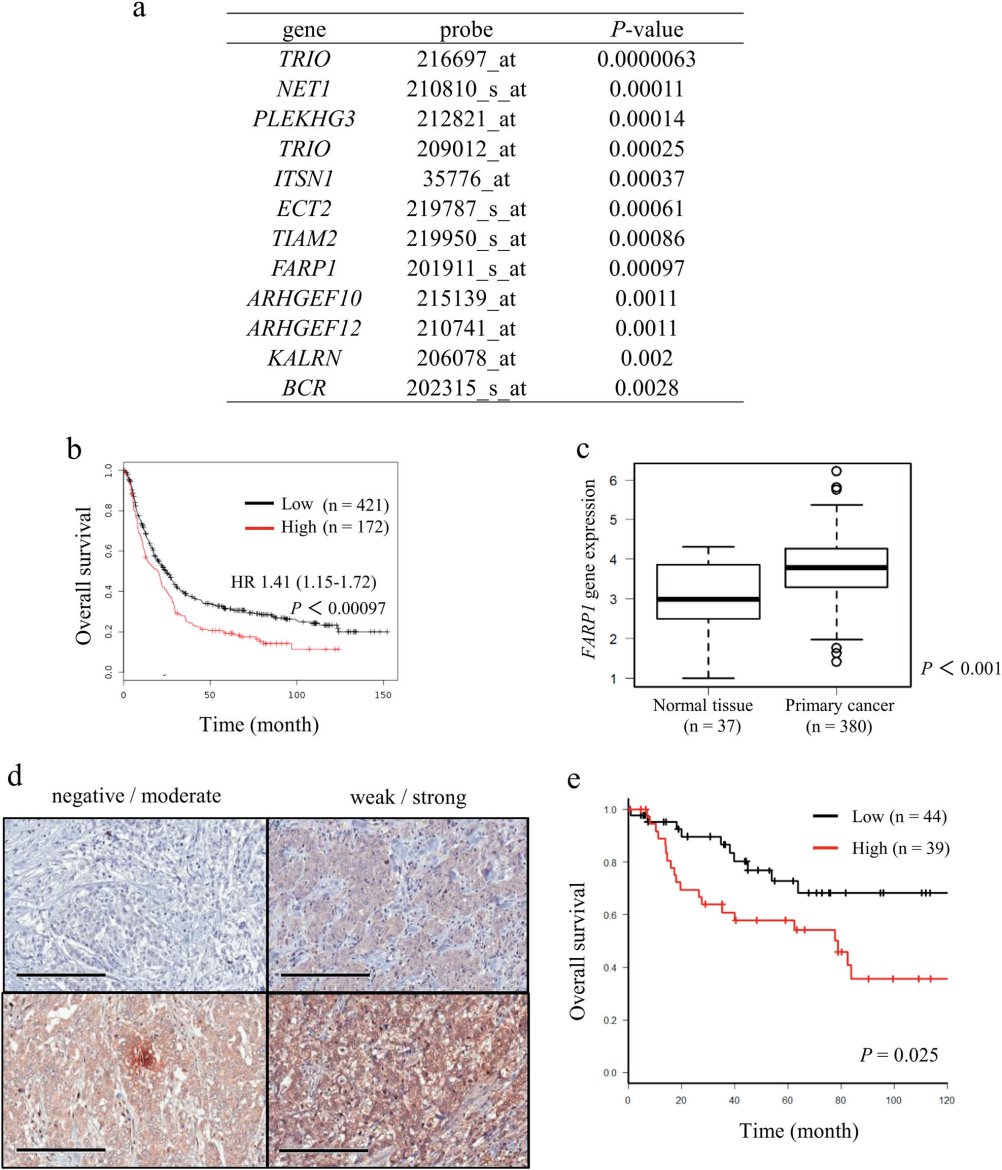
High expression of FARP1 is associated with poor prognosis in gastric cancer. **a** List of Rho GEF genes significantly correlated with poor prognosis of patients with gastric cancer. **b** Relationship between *FARP1* expression and overall survival of patients with gastric cancer as assessed using the Kaplan–Meier plotter. **c** Gene expression of *FARP1* in solid normal tissue and primary gastric cancer. Magnification, ×200; scale bar, 200 μm. **d** Intensity of anti-FARP1 staining in the cytoplasm of gastric cancer cells. **e** Overall survival of patients with gastric cancer within high and low FARP1 expression grouped according to immunohistochemistry assessment. Survival rates were calculated by the Kaplan–Meier method, and differences in survival were estimated by the log-rank test.

Kaplan–Meier analysis showed that high expression of 11 Rho GEF genes was significantly correlated with worse prognosis of patients with gastric cancer in GEO datasets (Fig. 1a). The Cancer Genome Atlas (TCGA) data analysis indicated that the gene expression of *TRIO*, *NET1*, *ECT2*, *TIAM2*, *FARP1*, *ARHGEF12* and *BCR* in primary cancer was significantly higher than those in normal tissues (Supplementary Fig. S1). Several investigators have previously reported the relevance of *TRIO*, *NET1*, *ECT2* and *TIAM2* in cancer metastasis and clinical prognosis^9,18–33^ (Supplementary Table 1). We further focused on *FARP1*, which has never been reported to have clinical significance in cancers. The prognostic value of *FARP1* expression in the Kaplan–Meier plotter and TCGA data analysis of *FARP1* expression in normal tissues and primary cancer are shown in Fig. 1b (HR 1.41 [1.15–1.72], *P* *=* 0.00097) and Fig.1c (*P* < 0.001), respectively.

### Correlation between FARP1 expression and clinicopathological findings in patients with advanced gastric cancer

To investigate whether the expression of FARP1 plays a role in gastric cancer development, we performed immunohistochemical analysis of 91 advanced gastric cancer samples (Fig. 1d). The accuracy of anti-FARP1 antibody was confirmed by immunohistochemical and immunofluorescence staining (Supplementary Fig. S2). The expression of FARP1 protein was associated with lymphatic metastasis (N) (*P* = 0.012), lymphatic invasion (ly) (*P* = 0.025) and recurrence rate (*P* = 0.002) but not with age, sex, pathological type, depth of invasion (T), pathological stage (pStage), venous invasion (v), or recurrence pattern (Table 1). The overall survival of patients in the high FARP1 expression group was significantly shorter than that in the low FARP1 expression group (*P* = 0.025) (Fig. 1e) in line with the in silico analysis.

**Table 1:** Correlation between FARP1 expression and clinicopathological factors in gastric cancer patients.

	FARP1 expression, *n* (%)		*P* value
	Low	High	
Patient, *n* = 91	47 (51.6)	44 (48.4)	
Age, *n* = 91			
≤65	20 (55.6)	16 (44.4)	0.697
<65	27 (49.1)	28 (50.9)	
Gender, *n* = 91			
Men	31 (50.0)	31 (50.0)	0.814
Women	16 (55.2)	13 (44.8)	
Adjuvant chemotherapy, *n* = 67			
Yes	20 (51.3)	19 (48.7)	0.269
No	19 (67.9)	9 (32.1)	
Pathological type, *n* = 91			
Differentiated	12 (42.9)	16 (57.1)	0.373
Undifferentiated	35 (55.6)	28 (44.4)	
T (pathological), *n* = 91			
pT2	9 (60.0)	6 (40.0)	0.654
pT3	23 (52.3)	21 (47.7)	
pT4a	15 (48.4)	16 (51.6)	
pT4b	0 (0.0)	1 (100.0)	
N (pathological), *n* = 91			
pN1	19 (82.6)	4 (17.4)	0.012
pN2	7 (43.8)	9 (56.2)	
pN3	8 (36.4)	14 (63.6)	
pN4a	8 (38.1)	13 (61.9)	
pN4b	5 (55.6)	4 (44.4)	
Stage, *n* = 91			
IB	8 (80.0)	2 (20.0)	0.352
IIA	8 (61.5)	5 (38.5)	
IIB	7 (58.3)	5 (41.7)	
IIIA	6 (37.5)	10 (62.5)	
IIIB	10 (50.0)	10 (50.0)	
IIIC	5 (35.7)	9 (64.3)	
IV	3 (50.0)	3 (50.0)	
Lymphatic invasion, *n* = 91			
ly1	14 (77.8)	4 (22.2)	0.025
ly2	16 (57.1)	12 (42.9)	
ly3	10 (43.5)	13 (56.5)	
ly4	7 (31.8)	15 (68.2)	
Venous invasion, *n* = 91			
v1	7 (41.2)	10 (58.8)	0.191
v2	23 (65.7)	12 (34.3)	
v3	9 (47.4)	10 (52.6)	
v4	8 (40.0)	13 (65.0)	
Recurrence, *n* = 82			
Yes	5 (22.7)	17 (77.3)	0.002
No	39 (65.0)	21 (35.0)	
Recurrence pattern, *n* = 20			
Local	1 (33.3)	2 (67.7)	0.886
Lymphogenus	1 (25.0)	3 (75.0)	
Hematogenous	1 (25.0)	3 (75.0)	
Peritoneal dissemination	0 (0.0)	3 (100.0)	
Multiple	1 (20.0)	4 (80.0)	
Follow-up lost, *n* = 7	3 (42.9)	4 (57.1)	1

Statistical analyses of two groups were performed using *χ*^2^ test.

### FARP1 expression promotes gastric cancer cell motility and promotes filopodium formation by activating CC42

Supplementary Fig. S3 shows the mRNA and protein FARP1 expression levels of the four human gastric cancer cell lines. Since MKN45 and MKN74 cells exhibited relatively higher endogenous FARP1 expression, *FARP1* RNA interference was performed in only these cells. The knockdown efficiency of siRNAs was confirmed by qPCR and western blot analysis (Supplementary Fig. S4a, b). Alternatively, MKN7 and GSU cells were infected with FLAG- enhanced green fluorescence protein (EGFP)- or FLAG-FARP1-expressing lentivirus, and the overexpression efficiencies of infection were confirmed by qPCR and western blot analysis (Supplementary Fig. S4c, d).

**Fig. 2:**
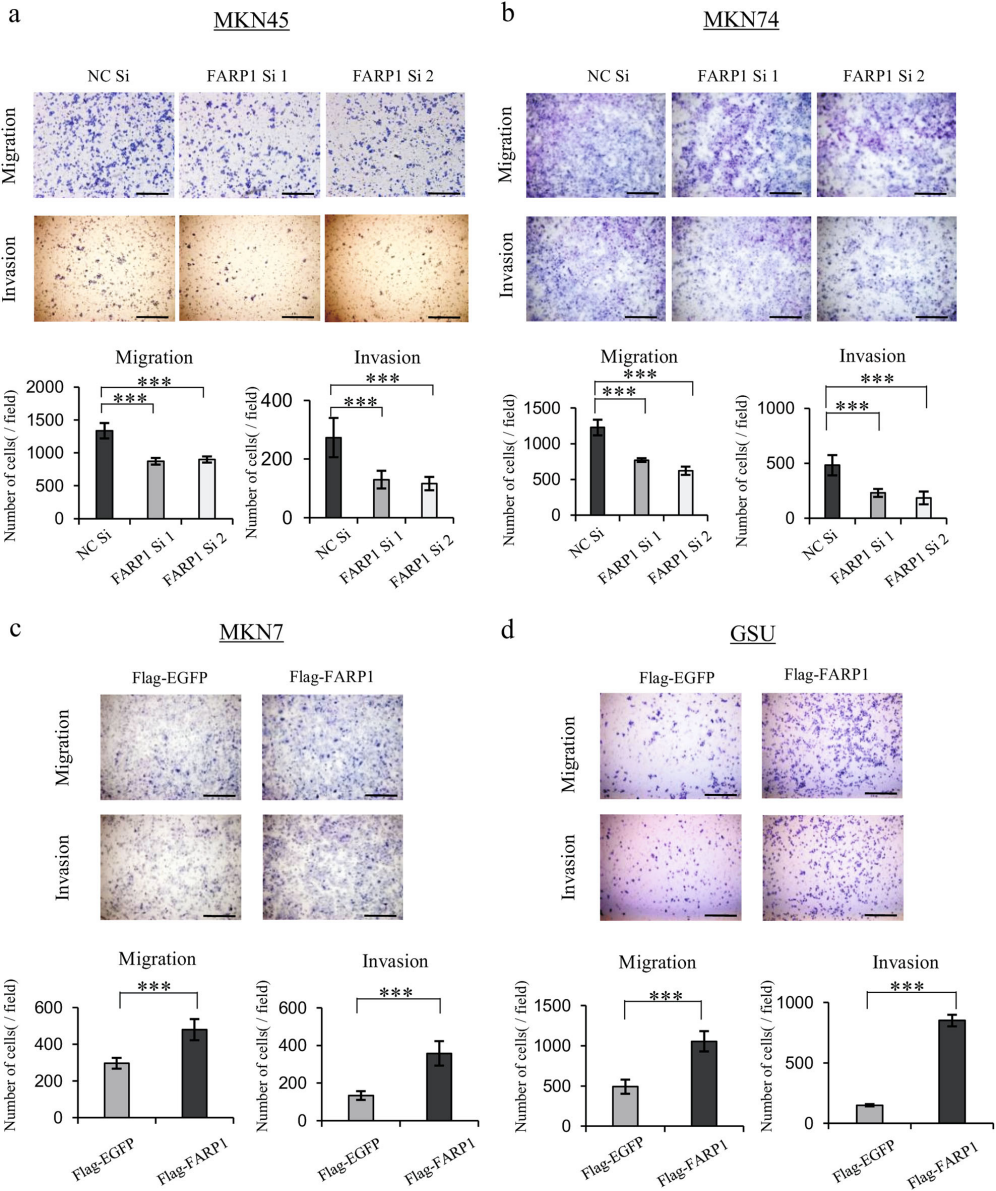
Effect of FARP1 expression on cell migration and invasion in gastric cancer cell lines. **a–d** Transwell migration and invasion assay in FARP1-knockdown (MKN45, MKN74) and FARP1-overexpressing (GSU, MKN7) cell lines. Magnification, ×100; scale bar, 500 μm. In (**a–d**), the graphs indicate the number of migratory and invasive cells. The values represent the means ± SD from six independent microscopic fields. ***P *<* 0.001 (Student’s *t* test).

The proliferation of FARP1-knockdown and FARP1-overexpressing cells was comparable to that of the control cells (Supplementary Fig. S5). FARP1 knockdown significantly decreased the numbers of migratory and invasive cells in both the MKN45 and MKN74 cell lines (Fig. 2a, b). Consistent with these findings, FARP1 overexpression significantly increased the numbers of migratory and invasive cells in the MKN7 and GSU cell lines (Fig. 2c, d).

**Fig. 3:**
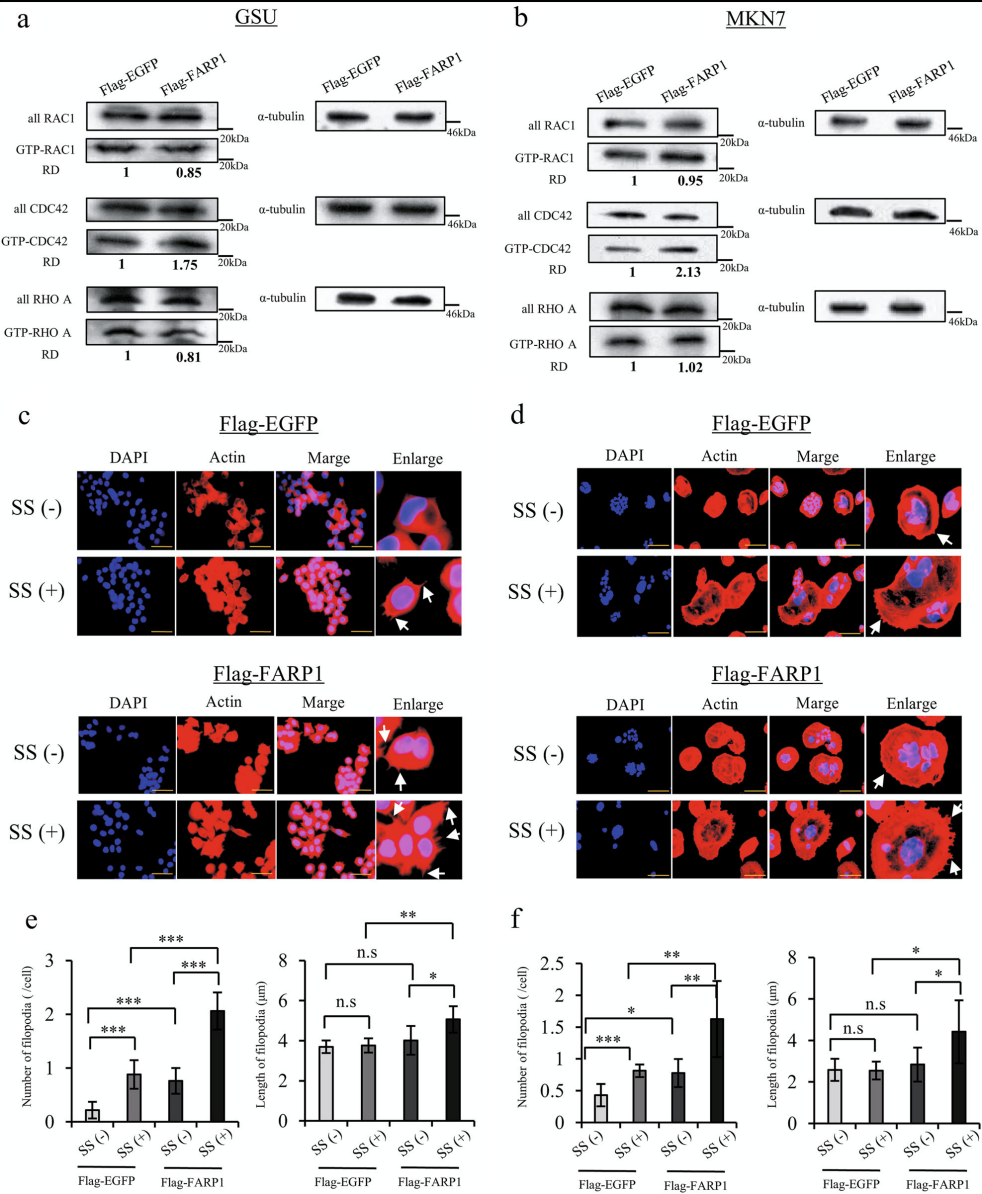
FARP1 activates CDC42 and promotes filopodium formation in gastric cancer cell lines. **a**, **b** Active Rac1/CDC42/RhoA pulldown assay with serum stimulation in GSU and MKN7 cells infected with the FLAG-EGFP- or FLAG-FARP1–expressing lentivirus. **c**, **d** Immunofluorescence staining for actin (red) and DAPI (blue) with or without serum stimulation in GSU and MKN7 cells infected with the FLAG-EGFP- or FLAG-FARP1-expressing lentivirus. Magnification, ×400; scale bar, 50 μm in each of the three photos; magnification, ×200; scale bar, 100 μm in enlarge. SS serum stimulation. White arrow, filopodium formation. **e**, **f** Number and length of filopodia in the FLAG-EGFP- or and FLAG-FARP1-expressing cells. Values represent the means ± SD from six independent fields. *P < 0.05, **P < 0.01, ***P < 0.001; n.s. not significant (Student’s *t* test); RD relative density.

Considering that Rho GEFs can directly activate Rho family proteins, we determined the amounts of activated RAC1, CDC42, and RHOA using a Rho small GTPase pulldown assay in FARP1-overexpressing cells upon serum stimulation. The amount of GTP-CDC42 increased in FARP1-overexpressing cells; however, the amount of GTP-RAC1 and GTP-RHOA in FARP1-overexpressing cells did not change (Fig. 3a, b). Conversely, the amount of GTP-CDC42 in FARP1-overexpressing GSU cells showed no distinct change compared with that of EGFP-overexpressing cells with no serum stimulation (Supplementary Fig. S6). Several investigators have reported that certain extracellular stimuli first activate Rho GEFs in a distinct manner^12,34,35^. Thus, these results may suggest that the FARP1-CDC42 cascade might be activated by particular extracellular signals.

In addition, as Rho family GTPases have important roles in the regulation of the cytoskeleton, we evaluated the effect of FARP1 expression on the cytoskeleton in GSU and MKN7 cell lines by detecting actin expression. Serum stimulation promoted filopodium formation in both EGFP- and FARP1-overexpressing cells, with FARP1-overexpressing cells exhibiting particularly greater filopodium formation than that of EGFP-overexpressing cells for both cell lines (Fig. 3c–f).

**Fig. 4:**
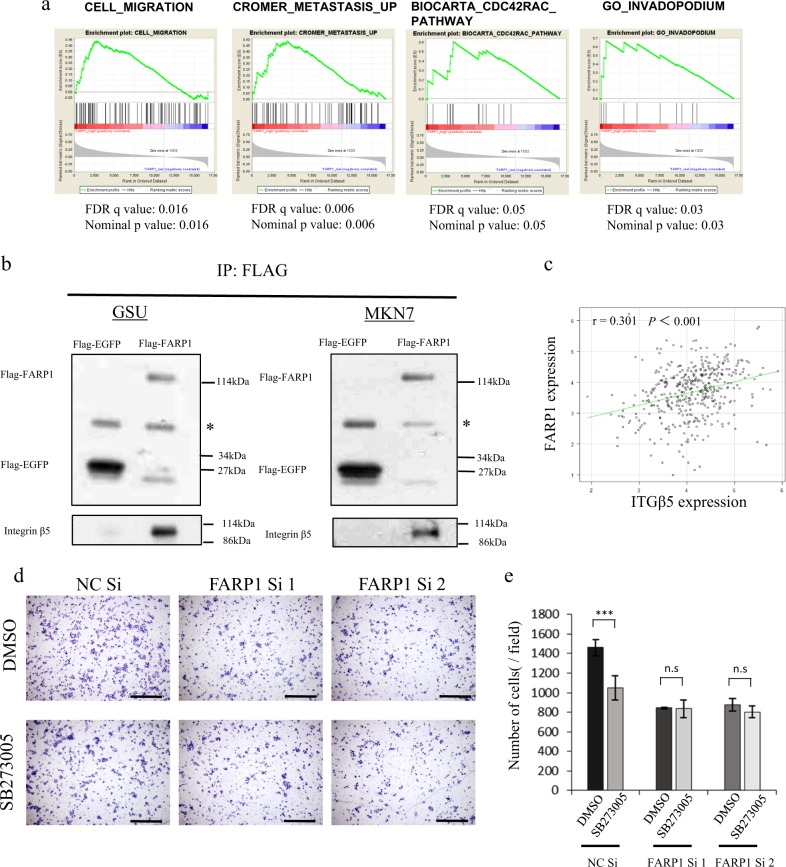
Interaction of FARP1 with integrin αvβ5 and expression of integrin β5 in gastric cancer. **a** Gene sets identified as being related to *FARP1* expression using GSEA. **b** Lysates from the FLAG-EGFP- or FLAG-FARP1-expressing GSU and MKN7 cells immunoprecipitated with the anti-FLAG antibody. Serum stimulation was applied before the cells were lysed. FLAG and integrin β5 were detected by western blotting. * indicate immunoglobulin heavy chain. **c** Correlation between *FARP1* mRNA expression and integrin *β5* mRNA expression in primary gastric cancer based on TCGA data. **d** Transwell migration assay in MKN74 cells transfected with NC Si, FARP1 Si1 or FARP1 Si2. **e** Numbers of migratory MKN74 cells transfected with NC Si, FARP1 Si1 or FARP1 Si2. In (**d**), 0.03% DMSO or 0.3 nM SB273005 diluted in 0.03% DMSO was applied when cells were inoculated onto the chamber. Magnification, ×100. Scale bar, 500 μm. **e** Values represent the means ± SD from six independent fields. ***P < 0.001; n.s. not significant (Student’s *t* test).

The gene expression and gene set enrichment analysis (GSEA) results of *FARP1* expression from TCGA data indicated that *FARP1* expression enriched the gene sets of CDC42 activation, migration, invadopodia, and metastasis (Fig. 4a), consistent with the in vitro results of FARP1 function.

### FARP1 interacts with integrin αvβ5, whereas inhibition of the integrin αvβ5 receptor decreases FARP1-induced filopodium formation and cell motility

Recently, several investigators reported the relationship of Rho GEFs and integrin^36,37^ and the direct interaction between FERM domain-containing proteins and integrin β5 was reported^38,39^. As FARP1 contains a FERM domain, we hypothesized that an interaction between FARP1 and integrin β5 might underlie the molecular mechanism of extracellular FARP1 activation. Immunoprecipitation with an anti-FLAG antibody followed by detection with an anti-integrin β5 antibody in FLAG-EGFP- or FLAG-FARP1-expressing GSU and MKN7 cells lysates indicated an interaction between FARP1 and integrin β5 (Fig. 4b). High *integrin β5* mRNA levels were associated with worse prognosis using the Kaplan–Meier plotter (HR 1.37 [1.11–1.68], *P* *=* 0.0029) (Supplementary Fig. S7a), and the gene expression level of *integrin β5* in primary gastric cancer was significantly higher than that in normal tissues based on TCGA data analysis (*P* *<* 0.001) (Supplementary Fig. S7b). Furthermore, a positive correlation was observed between *FARP1* mRNA expression and *integrin β5* mRNA expression (*P* < 0.001, *r* = 0.301) (Fig. 4c). These data support the existence of an integrin αvβ5-FARP1-CDC42 axis that promotes cancer development.

**Fig. 5:**
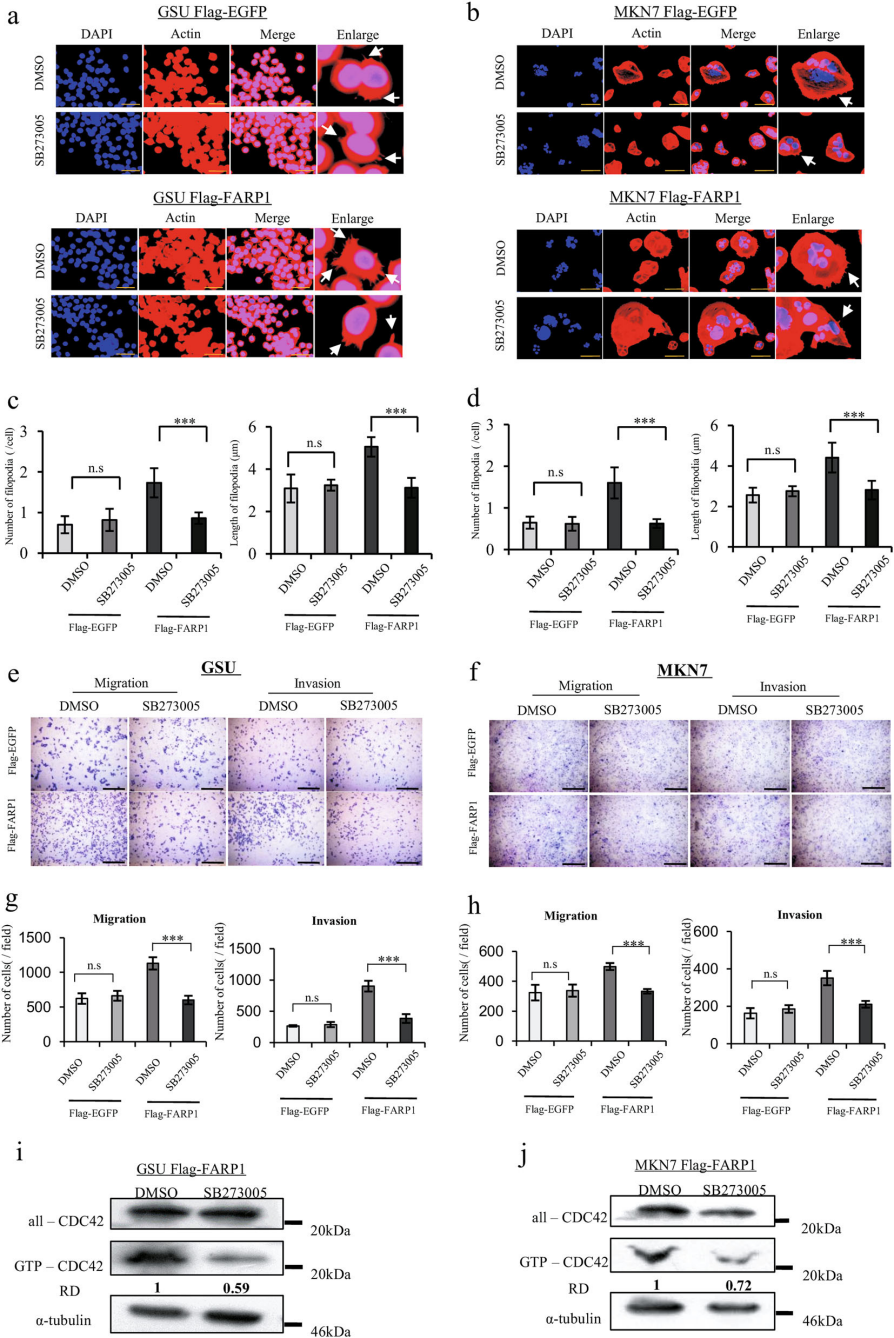
Inhibition of integrin αvβ5 receptor decreases FARP1-induced filopodium formation, cell motility, and CDC42 activation. **a**, **b** Immunofluorescence staining for actin (red) and DAPI (blue). **c**, **d** Number and length of filopodia of the FLAG-EGFP- or FLAG-FARP1-expressing GSU and MKN7 cells. **e**, **f** Transwell migration and invasion assay in the FLAG-EGFP or FLAG-FARP1-expressing GSU and MKN7 cells. **g**, **h** Numbers of migratory and invasive cells among the FLAG-EGFP- or FLAG-FARP1-expressing GSU and MKN7 cells. **i**, **j** Active CDC42 pulldown assay in GSU and MKN7 cells infected with the FLAG-EGFP- or FLAG-FARP1-expressing lentivirus. In (**a**, **b**, **i**, and **j**), the cells were starved in serum-free medium for 24 h and stimulated with 10% FBS for 2 h, and simultaneously, 0.03% DMSO or 0.3 nM SB273005 diluted in 0.03% DMSO was applied. White arrow, filopodia. Magnification, ×400; scale bar, 50 μm (**a**). Magnification, ×200; scale bar, 100 μm (**b**). In (**e** and **f**), 0.03% DMSO or 0.3 nM SB273005 diluted in 0.03% DMSO was applied when cells were inoculated onto the chamber. Magnification, ×100. Scale bar, 500 μm. **c**, **d**, **g**, and **h** Values represent the means ± SD from six independent fields. ***P < 0.001; n.s. not significant (Student’s *t* test); RD relative density.

To assess the importance of this interaction in gastric cancer cell motility, we used SB273005 (Selleck Chemicals, Houston, TX, USA), a potent integrin antagonist, with a *K*_i_ of 1.2 nM and 0.3 nM for the αvβ3 receptor and αvβ5 receptor, respectively. To minimize the effect of integrin αvβ3 inhibition, 0.3 nM SB273005 was used because higher concentrations have been reported to inhibit integrin αvβ3 as well. SB273005 significantly decreased the cell motility in control cells but did not change in FARP1-knockdown cells (Fig. 4d, e). In addition, SB273005 significantly decreased filopodium formation and cell motility in FARP1-overexpressing cells but not those in EGFP-overexpressing cells (Fig. 5a–h). Moreover, SB273005 decreased the amount of GTP-CDC42 in FARP1-overexpressing GSU and MKN7 cells (Fig. 5i, j). These results consistent with that integrin αvβ5 signaling enhances to activate FARP1.

## Discussion

The roles of FARP1 expression in cancer development are not well understood. In this study, we showed that FARP1 overexpression was significantly associated with lymphatic invasion, lymph metastasis, and poor prognosis in patients with advanced gastric cancer, and that it promoted gastric cancer cell motility by activating CDC42. FARP1 was recently reported to activate CDC42 in the endothelial barrier^17^, and a correlation between CDC42 activity and FARP1 expression was identified in pheochromocytoma^40^. FARP1 was also reported to specifically activate Rac1 in dendrites^16^ and to be able to activate RhoA, as demonstrated using an Sf9-overexpressing system^41^. In turn, CDC42 has been considered to contribute to a variety of cellular responses, including cellular transformation, cell division, cell migration, cell invasion, filopodium formation, invadopodium formation, enzyme activity, and cell polarity^42^. Thus, it appears reasonable to conclude that FARP1 activates CDC42 and facilitates the abilities of cell migration and invasion by promoting the formation of filopodia and invadopodia in gastric cancer. The GSEA results were consistent with this conclusion. Zhou et al.^43^ also recently reported the clinical significance of FARP1 in gastric cancer using in silico analysis, which is consistent with our results. Shannon et al.^20^ reported that two GEF-GTPase signaling units, ECT2-CDC42 and TRIO-RAC1, involved in glioblastoma cell migration and invasion. A positive correlation was observed between *FARP1*mRNA expression and *TRIO*, *PLEKHG3*, *ITSN1*, *TIAM2*, *ARHGEF10* and *ARHGEF12* mRNA expression based on TCGA data analysis and *TRIO* showed the most strongest correlation value (*P* = 0, *r* = 0.426) (Supplementary Fig. S8). Therefore, FARP1 and TRIO may involve in gastric cancer cell migration, invasion and poor prognosis in a synergistic manner.

The integrin family, which consists of 24 heterodimeric transmembrane receptors, mediates the interaction between cells and extracellular matrices and is involved in cell adhesion and migration. Several integrin heterodimers have already been reported to be involved in gastric cancer biology^44–46^. For example, integrin αvβ5 was previously recognized as a putative target for the treatment of several cancers^47–49^. Recently, the efficacy of cilengitide, a potent and selective inhibitor of integrins αvβ3 and αvβ5, in combination with cytotoxic agents has been reported^50,51^. However, only two studies have focused on integrin αvβ5 in gastric cancer, and its roles in the development of gastric cancer remain controversial^52,53^. Our in silico analysis indicated that high mRNA expression levels of integrin β5 were correlated with a poor prognosis of patients with gastric cancer; moreover, we provided the first demonstration of the interaction between FARP1 and integrin αvβ5 in gastric cancer cell lines. In addition, we found that inhibition of the integrin αvβ5 receptor significantly decreased the cell motility capability in high FARP1-expressing gastric cancer cells. This result suggests that inhibition of the FARP1-integrin αvβ5 pathway might improve the survival of patients with high FARP1-expressing gastric cancer.

Although MAP4K4 has been reported to phosphorylate FARP1, the activation mechanisms of FARP1 are poorly understood^54,55^. FARP1 and its close homolog FARP2 contain an FERM domain, a DH domain, and two PH domains (PH1 and PH2), and share a high degree of sequence identity, excluding the FERM/DH linker. Although their DH-PH-PH domains have abundant tyrosine residues at the PH2/DH interface, crystal structural analysis suggests that an autoinhibitory mechanism by the C terminal portion of the sixth helix, which contains no tyrosine residues in the DH domain, can inhibit phosphorylation mediated by Src or other kinases. For this reason, tyrosine phosphorylation alone appears to be insufficient for triggering the full activation of FARPs^56^. Alternatively, it was recently reported that the recruitment of FARP1 to the membrane, induced by semaphorin 3A/PlexinA1 signaling combined with the plexinA1–FARP1 interaction, regulates FARP1 activity in dendrites^57^. Therefore, the suppression of an autoinhibitory mechanism through the conformational change effected by interacting with integrin αvβ5 and phosphorylation by EGFR-MAP4K4 signaling might, in turn, regulate FARP1 activity in gastric cancer cells.

However, the minimum amount of SB273005 required for integrin αvβ5 inhibition was used in this study; therefore, it is likely that SB273005 also affected integrin αvβ3 and gastric cancer cell motility. It has been reported that low (nanomolar) concentrations of the RGD mimetics αvβ3 and αvβ5 cause alterations in integrin αvβ3 and, paradoxically, promote tumor growth and angiogenesis^58^. This might explain why SB273005 had no impact on the motility of EGFP-overexpressing gastric cancer cells. Therefore, to evaluate the actual inhibitory effect in relation to FARP1 and integrin αvβ5 interaction, additional experiments using specific inhibitors for FARP1-integrin αvβ5 interaction are required.

Overall, this study shows that FARP1 interacts with integrin αvβ5 and promotes cell motility through the activation of CDC42. This is consistent with the observation that the overexpression of FARP1 protein correlates with unfavorable prognosis in patients with advanced gastric cancer. This study is based on a limited number of clinical samples and in vitro experiments using gastric cancer cell lines. Although to confirm the clinical relevance of the findings of this study and the molecular mechanism of FARP1, we must analyze more samples and in vivo experiments, our findings suggest that FARP1 may represent a crucial marker to predict the prognosis of patients with gastric cancer and that the integrin αvβ5-FARP1-CDC42 pathway may serve as a target for molecular therapy in these patients. Thus, regulatory cascade upstream of Rho can be a specific and promising target of advanced cancer treatment.

## Materials and methods

### In silico analysis to determine the relationship between Rho GEF expression and patient prognosis

Comprehensive analyses and multiple testing corrections at a false discovery rate (FDR) of 10% were performed for 72 Rho GEFs in 593 patients with gastric cancer from GEO datasets using the Kaplan–Meier plotter (http://kmplot.com/analysis/) to evaluate the relationship between Rho GEF expression and prognosis^59^. We used the default settings (except for GSE62254) according to the software developer’s recommendation. To correct the *P* value for multiple probes, we used the “multiple hypothesis testing” option, available at the same site, to acquire *q* values for the FDR.

### Drugs, reagents, and antibodies

The following reagents were purchased from the indicated manufacturers: MTT (3-(4,5-dimethylthiazol-2-yl)-2,5-diphenyl tetrazolium bromide) (Sigma-Aldrich); monoclonal antibodies against FARP1 (Novus Biologicals Agent, Cat#H00010160-M01), alpha tubulin (Millipore, Cat# CP06), FLAG (Sigma-Aldrich, Cat# F1804), integrin β5 (Cell Signaling Technology, Cat# 4708P), RHOA (Cell Signaling Technology, Cat# 2117), CDC42 (Cell Signaling Technology, Cat# 2466), and RAC1 (packed in RHOA/ RAC1/CDC42 Activation Combo Kit, Cell Biolabs, Cat# STA-405); SB273005 (a potent integrin inhibitor with *K*_i_ of 1.2 and 0.3 nM for αvβ3 receptor and αvβ5 receptor, respectively) (Selleck Chemicals).

### Patients and tumor samples

This study included 91 consecutive patients with advanced gastric cancer who underwent gastrectomy at the Department of Digestive Surgery, Breast and Thyroid Surgery of Kagoshima University Hospital from April 2002 to March 2011 (Table 1). Patients who received neoadjuvant chemotherapy or had remnant gastric cancer and multiple primary cancers were excluded. The clinical samples were obtained from tumors removed during surgery and ultimately diagnosed as gastric cancer pathologically. The pathological features of gastric cancer were classified according to the TNM classification, seventh edition^60^.

### Immunohistochemical analysis and capture of histological images

The surgical samples were fixed in 10% formaldehyde and embedded in paraffin before being cut into 3-µm-thick slices. Deparaffinization, hydrophilization, and target retrieval were performed using the PT Link system (Dako, Glostrup, Denmark). Endogenous peroxidase activity was blocked using 3% hydrogen peroxide in methanol. After the sections were washed with PBS, they were preincubated in 1% bovine serum albumin for 30 min to block nonspecific reactions at room temperature. The sections were incubated with the FARP1 mouse monoclonal antibody (1:400 dilution) as the primary antibody overnight at 4 °C. Staining was performed using the avidin–biotin complex and immunoperoxidase method (Vectastatin ABC Kit, Vector Laboratories, Burlingame, CA, USA). The sections were visualized using diaminobenzidine tetrahydrochloride, and nuclei were stained with hematoxylin. The images of the specimens were obtained using an Aperio CS2 scanner (Leica Biosystems, Wetzlar, Germany).

### Evaluation of FARP1 protein expression

FARP1 staining was performed in the most invasive portion of tumors and observed across five microscopic fields (magnification, ×200). The expression level of FARP1 was scored according to the intensity of cytoplasmic staining as negative (0), weak (1), moderate (2), or strong (3) (Fig. 1d), and the percentage of stained tumor cells was scored as 0% (0), 1–10% (1), 11–50% (2), 51–80% (3), or 81–100% (4). Scores were multiplied to obtain the immunoreactivity score (IRS), ranging from 0 to 12, as described previously^61^. The IRS value were evaluated independently by two board-certified pathologists those are unaware of clinical data. The accordance was 86/91 (94.5%) and the inconsistent cases were re-evaluated by the two pathologists under agreement (I.K. and A.T). Patients were divided into two groups of high or low FARP1 expression based on the median IRS value.

### Cell lines and culture

To investigate the molecular role of FARP1 in the development of gastric cancer, we used four gastric cancer cell lines. The human gastric cancer cell lines MKN7 (RCB Cat# RCB0999, RRID: CVCL_1417), MKN45 (RCB Cat# RCB1002, RRID: CVCL_2791), MKN74 (RCB Cat# RCB1001, RRID: CVCL_0434), and GSU (RCB Cat# RCB2278, RRID: CVCL_8877) were obtained from RIKEN BioResource Center (Tsukuba, Japan). The cells were cultured in RPMI-1640 medium (Sigma-Aldrich) supplemented with antibiotics (100 U/mL penicillin) and 10% FBS (Thermo Fisher Scientific, Waltham, MA, USA). All cancer cell lines were cultured at 37 °C in a humidified atmosphere containing 5% CO_2_.

### RNA isolation and reverse transcription-quantitative (q)PCR

Total RNA from the cultured cells was isolated using TRIzol (Molecular Research Center, Cincinnati, OH, USA) and reverse-transcribed using the ReverTra Ace kit (Toyobo, Oosaka, Japan), according to the manufacturer’s instructions^62^.

The mRNA expression levels of *FARP1* were determined by qPCR on the Step One Plus system (Applied Biosystems) with the forward primer 5′-CATTCTATCCGGAGCCTTGC-3′ and the reverse primer 5′-GGAACCTTCGGTTCCTTTCC-3′ using GoTaq qPCR Master Mix (Promega, Madison, WI, USA), according to the manufacturer’s instructions. Human *GAPDH* was used for normalization with the forward and reverse primers 5′-TGCACCACCAACTGCTTAG-3′ and 5′-GAGGCAGGGATGATGTTC-3′, respectively. The expression of the target gene was quantified using the comparative cycle threshold method. The primers were synthesized by FASMAC (Kanagawa, Japan).

### Protein extraction and immunoblotting

The total cell lysate was isolated using RIPA buffer (25 mM Tris-HCl pH 7.5, 150 mM NaCl, 1% Nonidet P-40, 0.1% SDS, 0.5% sodium deoxycholate) and a proteinase inhibitor cocktail (Nacalai Tesque, Kyoto, Japan). Protein concentrations were measured using Protein Assay CBB Solution (5×) (Nacalai Tesque). Cell lysates were separated by 5–20% SDS-PAGE (ATTO, Amherst, NY, USA) and transferred onto polyvinylidene fluoride membranes. The blotted membranes were incubated with anti-FARP1 (1:750 dilution), anti-alpha-tubulin (1:1000 dilution), anti-FLAG (1:1000 dilution), anti-RAC1 (1:1000 dilution), anti-CDC42 (1:500), anti-RHOA (1:500), or anti-integrin β5 (1:750 dilution) antibody overnight at 4 °C, and each protein was detected as described previously^63^.

### siRNA transfection

FARP1 siRNA1 (5′-CAAAUUUCAUACUAAUUUU-3′) and siRNA2 (5′-CCUUCUUUAGACUUUUUGA-3′) were synthesized by FASMAC. FARP1 siRNA1, FARP1 siRNA2, and Silencer Negative Control No. 1 siRNA (NC Si) (Thermo Fisher Scientific) were transfected to MKN45 and MKN74 cells at 50 nM each using Lipofectamine RNAiMAX (Thermo Fisher Scientific) in serum-free medium for 24 h.

### FARP1 lentivirus expression vector construct

Full-length *FARP1* open reading frame (ORF) cDNA along with a FLAG Tag was obtained from MKN45 cells by reverse transcription-qPCR with the forward primer 5′-TAGCTAGCACCATGGACTACAAGGACGACGATGACAAGGGAGAAATAGAGCAGAGGCC-3′ and reverse primer 5′-TAGCGGCCGCTCAATACACA AGAGACTCTT-3′ synthesized by FASMAC. PCR products were purified and confirmed by DNA sequencing. The EGFP ORF was acquired from pEGFP-C2 (Clontech Laboratories, Mountain View, CA, USA). These cDNAs, along with a FLAG Tag, were ligated into CSII-CMV-MCS-IRES2-Bsd (RIKEN BioResource Center). FARP1 and EGFP expression recombinant lentiviruses were produced by cotransfection of 293T cells with CSII-CMV-MCS-IRES2-Bsd-FLAG-FARP1 or CSII-CMV-MCS-IRES2-Bsd-FLAG-EGFP, together with the lentivirus packaging plasmids pMDLg/pRRE, pRSV-REV, and pMD2.G (Addgene, Cambridge, MA, USA) using Lipofectamine 2000 (Thermo Fisher Scientific). MKN7 and GSU cells were infected with the lentivirus for 48 h and then incubated with 5 µg/mL blasticidin S hydrochloride (Kaken Pharmaceuticals, Tokyo, Japan) for at least 5 days.

### Cell proliferation assay

Equal numbers of cells (1 × 10^3^) were seeded into 96-well plates and incubated for 1, 3, 5, and 7 days. Cell viability was measured using the MTT colorimetric assay^64^. These experiments were performed independently at least three times.

### Transwell migration and invasion assay

BioCoat Control Inserts and BioCoat Matrigel Invasion Chamber (24-well, 8 µm; Corning, Corning, NY, USA) were used for cell migration and invasion assays. Chamber membranes of the control inserts were coated with 10 µg/mL fibronectin (Sigma-Aldrich). Cells were inoculated with serum-free medium into the top chamber, and the bottom chamber was filled with medium containing 10% FBS and 1 ng/mL epidermal growth factor (PeproTech, Rocky Hill, NJ, USA). The numbers of inoculated cells were as follows: MKN45, 3.0 × 10^5^; MKN74, 1.5 × 10^5^; MKN7, 1.5 × 10^5^; and GSU, 1.0 × 10^5^. MKN74 and GSU cells were incubated for 48 h, and MKN45 and MKN7 cells were incubated for 72 h. After incubation, the bottom membranes were fixed using 4% paraformaldehyde and stained with hematoxylin. Cell numbers were counted in six fields under a microscope. These experiments were performed independently at least three times.

### Rho small GTPase pulldown assay

Cells were incubated until they reached approximately 80–90% confluence, after which they were starved in serum-free medium for 24 h and stimulated with 10% FBS for 2 h (serum stimulation) as described previously^10^. The total cell lysate was isolated using RIPA buffer without SDS. Cell lysates were incubated with PAK1 RBD or Rhotekin PBD agarose beads (Cell Biolabs) for 1 h at 4 °C. GTP-RAC1, GTP-CDC42, and GTP-RHOA were detected by immunoblotting. Densities of the individual bands were quantified using ImageJ software (RRID: SCR_003070; National Institutes of Health). The densities of GTP-form bands were normalized to the densities of bands of each total Rho family protein. Relative densities (RDs) were obtained by comparisons with the density of each GTP-form band of FLAG-EGFP–expressing cells.

### Immunoprecipitation

The total cell lysate was isolated using RIPA buffer without SDS. Cell lysates were incubated with anti-FLAG antibody, followed by COSMOGEL (R) Ig Accept Protein G (Nacalai Tesque) for 1 h at 4 °C. FLAG and integrin β5 were detected by immunoblotting.

### Immunofluorescence

Cells (1.0 × 10^4^) were seeded into a four-chamber CELLview cell culture dish (Greiner Bio-One, Kremsmuenster, Austria). The cells were fixed with 4% paraformaldehyde for 10 min at room temperature, followed by permeabilization with PBS containing 1% bovine serum albumin and 0.3% Triton X-100. The cells were subjected to immunofluorescence staining for actin using ActinRed 555 ReadyProbes (Thermo Fisher Scientific) for 30 min at room temperature and then incubated with DAPI (Dojindo Laboratories, Kumamoto, Japan) (1:4000 dilution) for 10 min at room temperature. The cells were subjected to immunofluorescence staining for FARP1 using anti-FARP1 antibody (1:200 dilution) for 1 h at room temperature, followed by treating with Alexa Fluor 647 (Abcam, Cambridge, UK, Cat# ab150115) (1:500 dilution) as secondary antibody for 1 h at room temperature and then incubated with DAPI for 10 min at room temperature. The images of cells were obtained using Axio Observer Z1 (Carl Zeiss, Oberkochen, Germany). The number and length of filopodia over 1 μm were quantified using ImageJ software in six fields.

### GSEA in gastric cancer data from TCGA

TCGA stomach cancer RNA-Seq (level 3) data, recorded as log2(*x* + 1) transformed read per kilobase of exon per million mapped reads (RPKM) values, were downloaded from UCSC Xena (http://xena.ucsc.edu). The gene expression levels of *FARP1* and integrin β5 in solid normal tissue and primary tumor were compared and correlation for these gene expression level in primary tumor was generated using Pearson’s correlation coefficient. GSEA was performed using GSEA v3.0 (Broad Institute, Cambridge, MA, USA). The *FARP1* expression level was divided into low and high categories to annotate phenotype, and gene sets (CELL_MIGRATION, CROMER_METASTASI_UP, BIOCARTA_CDC42RAC_PATHWAY, and GO_INVADOPODIUM) from Molecular Signature Database v6.1 (http://software.broadinstitute.org/gsea/msigdb/index.jsp) were used. All other parameters were set based on their default values^65^. An FDR *q* value < 0.25 or nominal *P* value < 0.05 was considered statistically significant.

### Statistical analysis

All statistical calculations were carried out using EZR^66^. Statistical analyses of group differences were performed using the *χ*^2^ test with Yate’s continuity correction and unpaired, two-sided Student’s *t* test. Kaplan–Meier survival curves were generated to compare the high and low FARP1 expression groups using the log-rank test. *P* values of <0.05 were considered statistically significant. Error bars represent standard deviations.

## Supplementary information

Supplementary Table 1

Supplementary Fig.S1

Supplementary Fig.S2

Supplementary Fig.S3

Supplementary Fig.S4

Supplementary Fig.S5

Supplementary Fig.S6

Supplementary Fig.S7

Supplementary Fig.S8
