# Supplementary figures and images for: FARP1 boosts CDC42 activity from integrin αvβ5 signaling and correlates with poor prognosis of advanced gastric cancer
Source: Oncogenesis. 2020 Feb 6;9(2):13. doi: 10.1038/s41389-020-0190-7 (PMC7005035; doi:10.1038/s41389-020-0190-7)

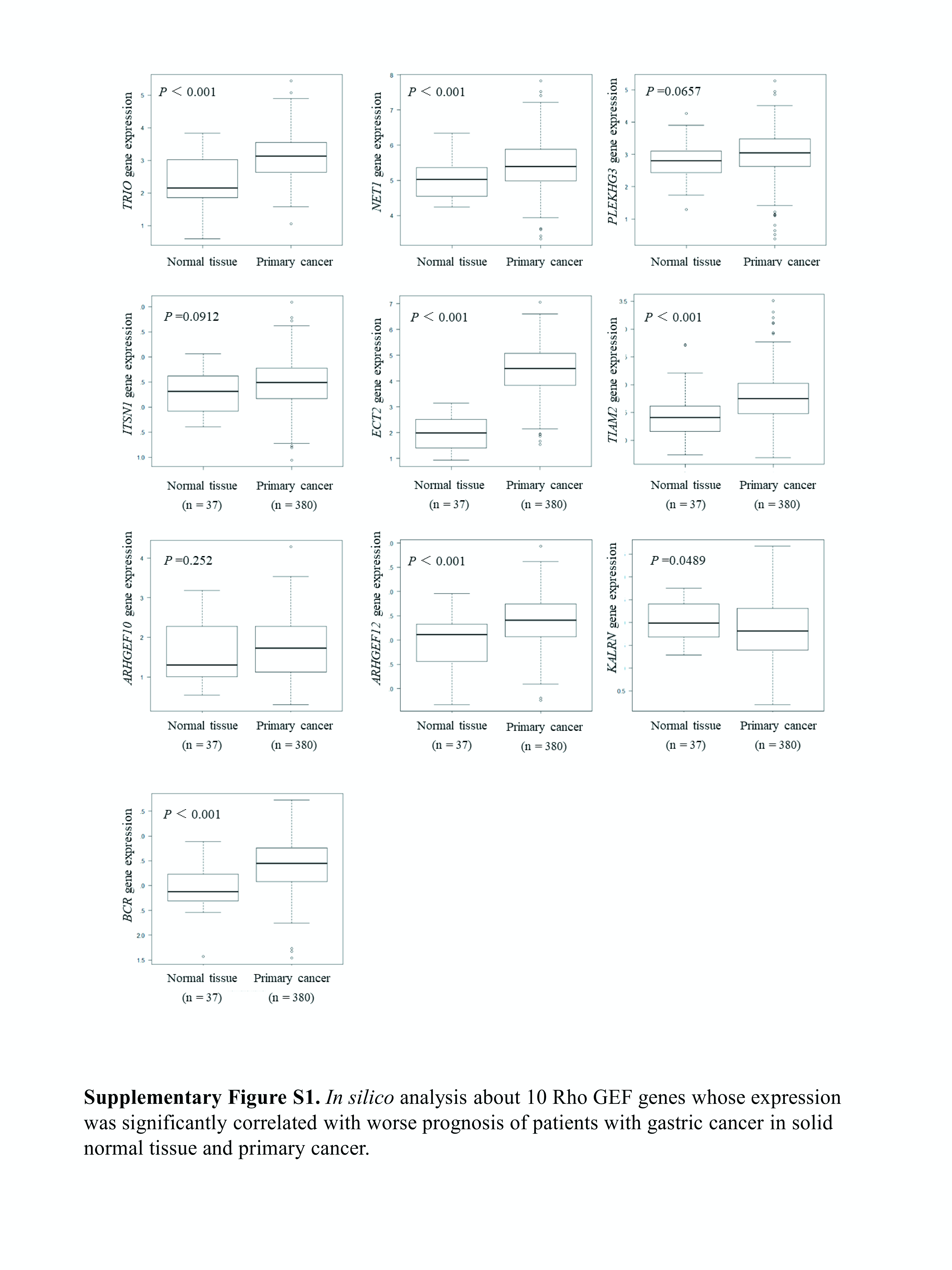

Supplement: Supplementary file 2 — Supplementary Fig.S1 [file 41389_2020_190_MOESM2_ESM.tif]

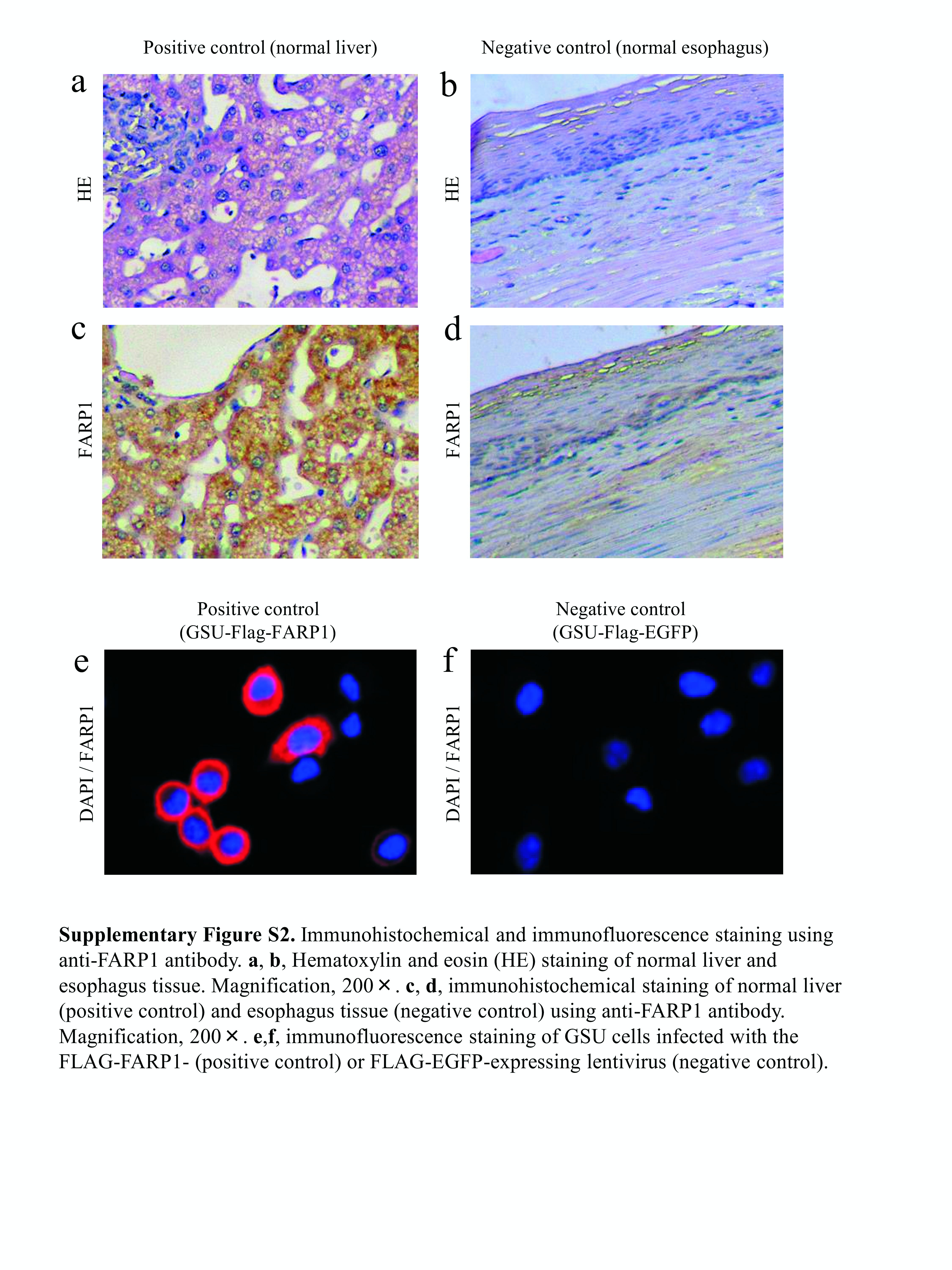

Supplement: Supplementary file 3 — Supplementary Fig.S2 [file 41389_2020_190_MOESM3_ESM.tif]

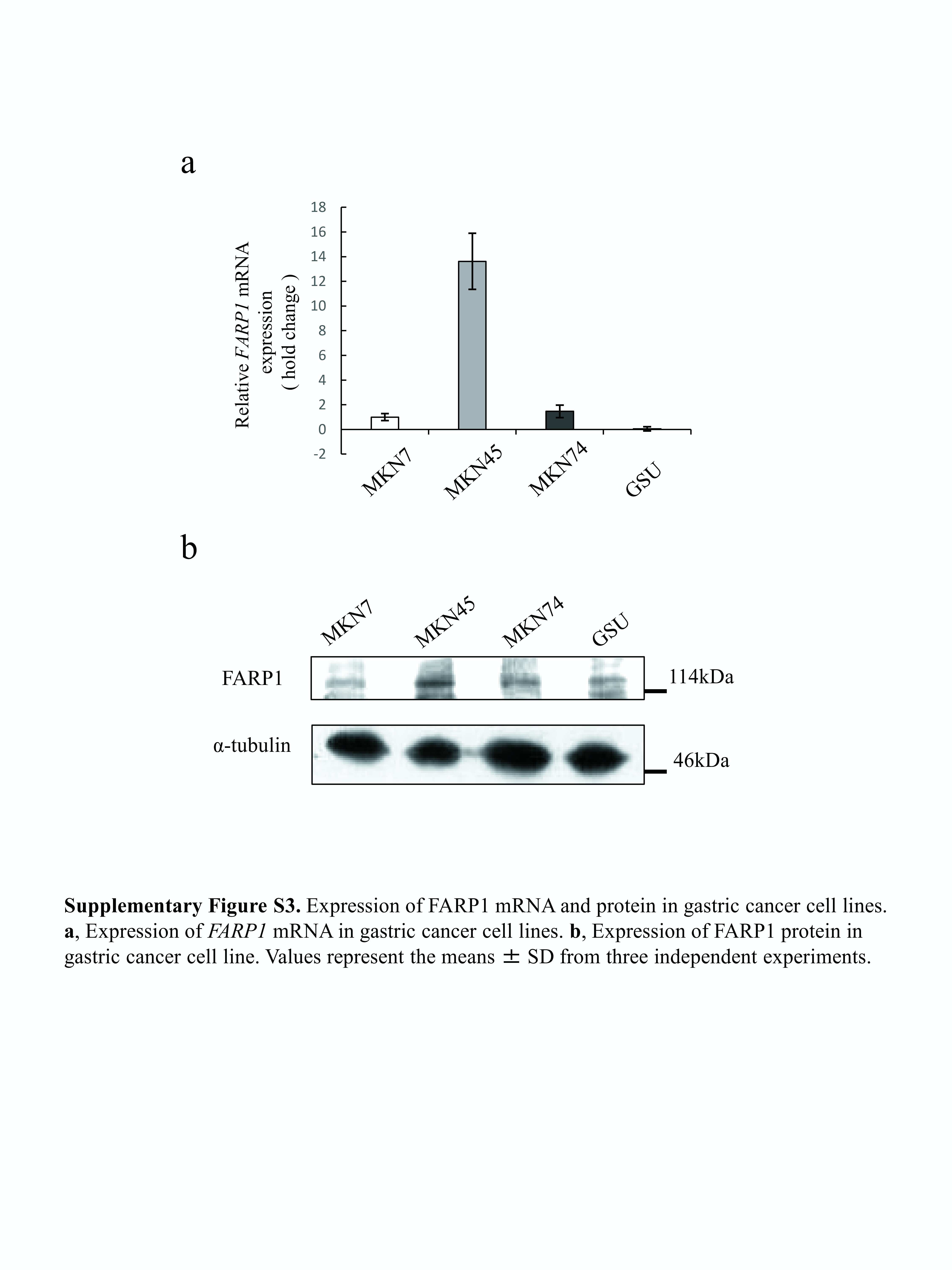

Supplement: Supplementary file 4 — Supplementary Fig.S3 [file 41389_2020_190_MOESM4_ESM.tif]

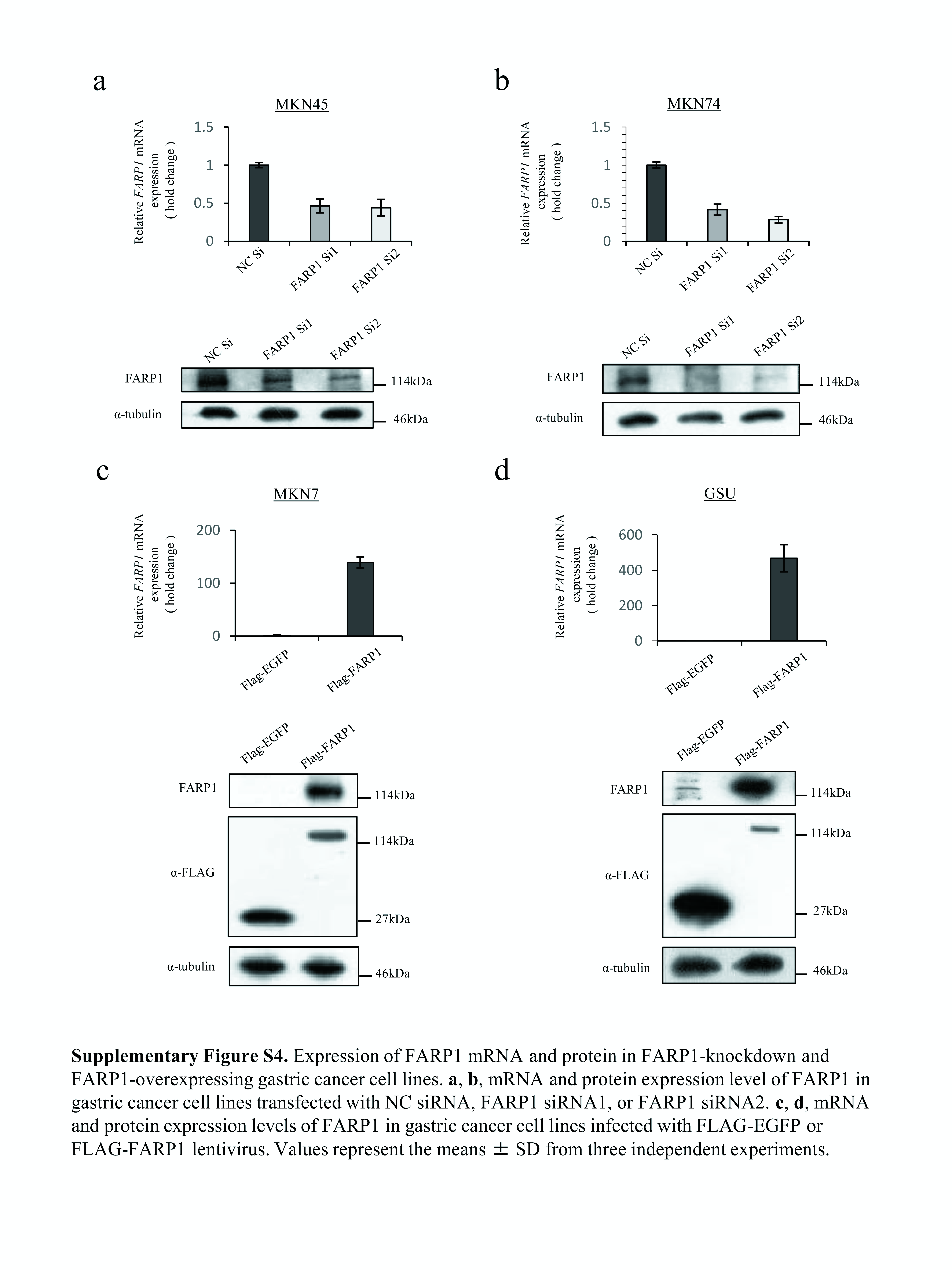

Supplement: Supplementary file 5 — Supplementary Fig.S4 [file 41389_2020_190_MOESM5_ESM.tif]

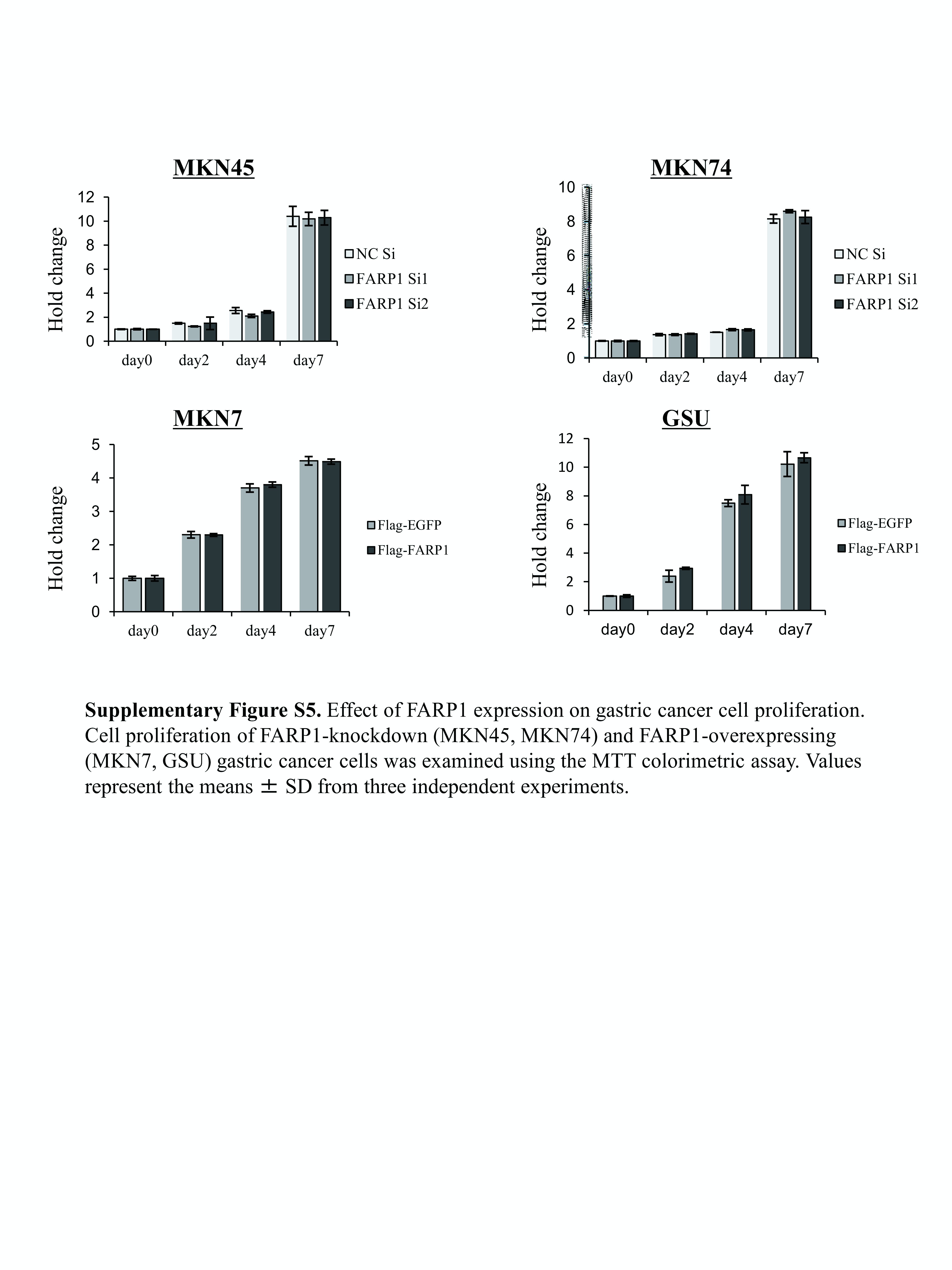

Supplement: Supplementary file 6 — Supplementary Fig.S5 [file 41389_2020_190_MOESM6_ESM.tif]

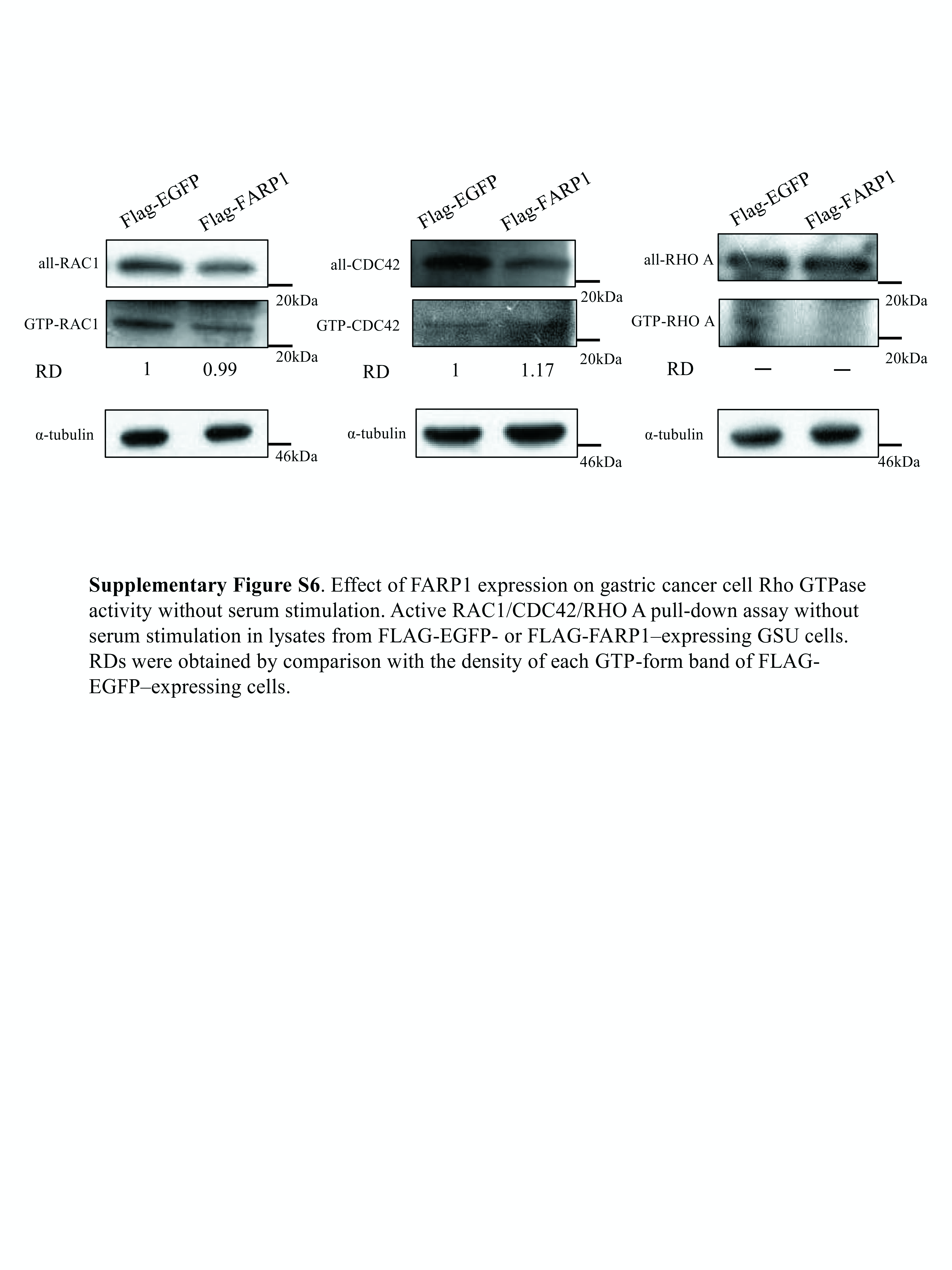

Supplement: Supplementary file 7 — Supplementary Fig.S6 [file 41389_2020_190_MOESM7_ESM.tif]

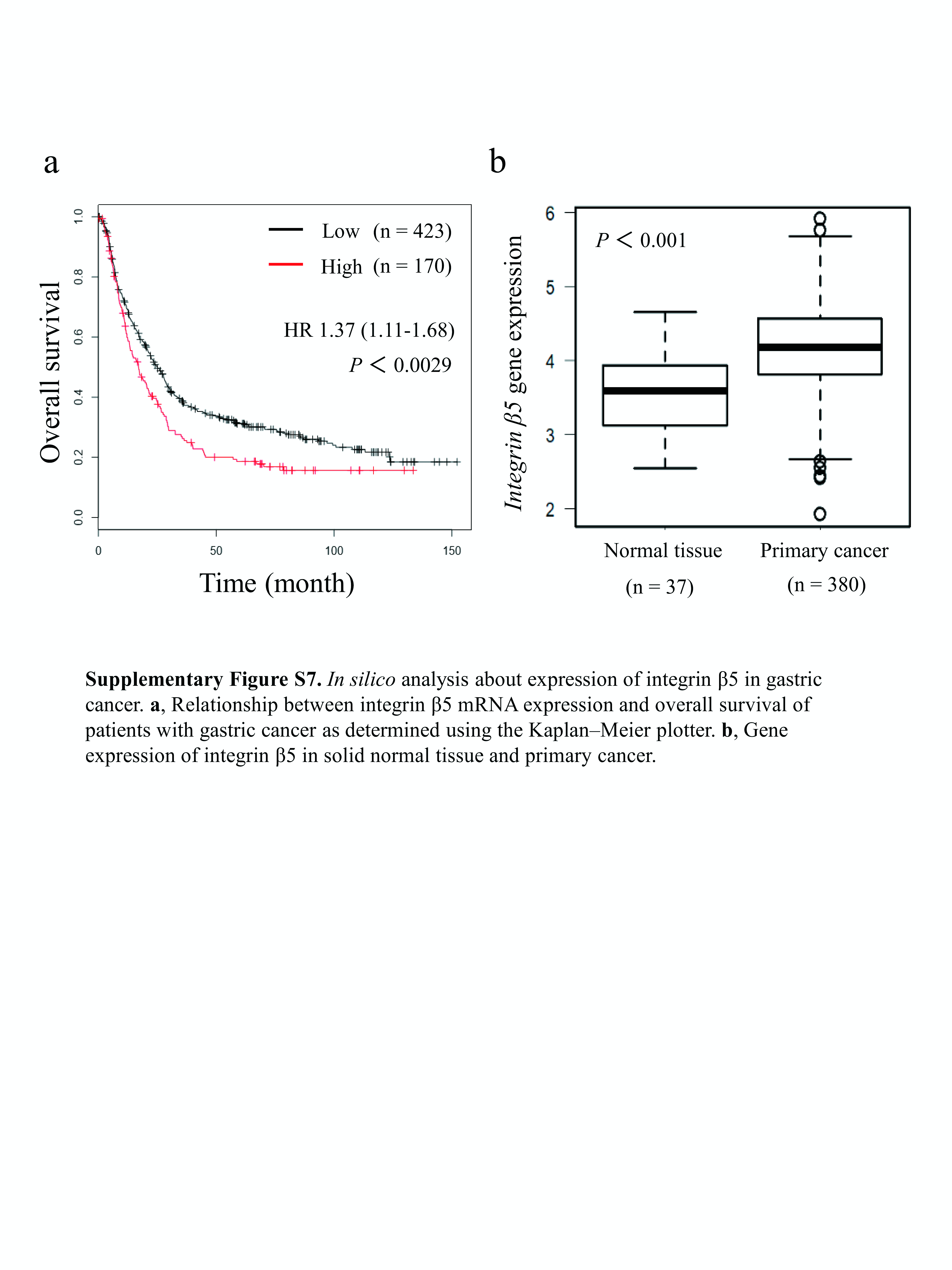

Supplement: Supplementary file 8 — Supplementary Fig.S7 [file 41389_2020_190_MOESM8_ESM.tif]

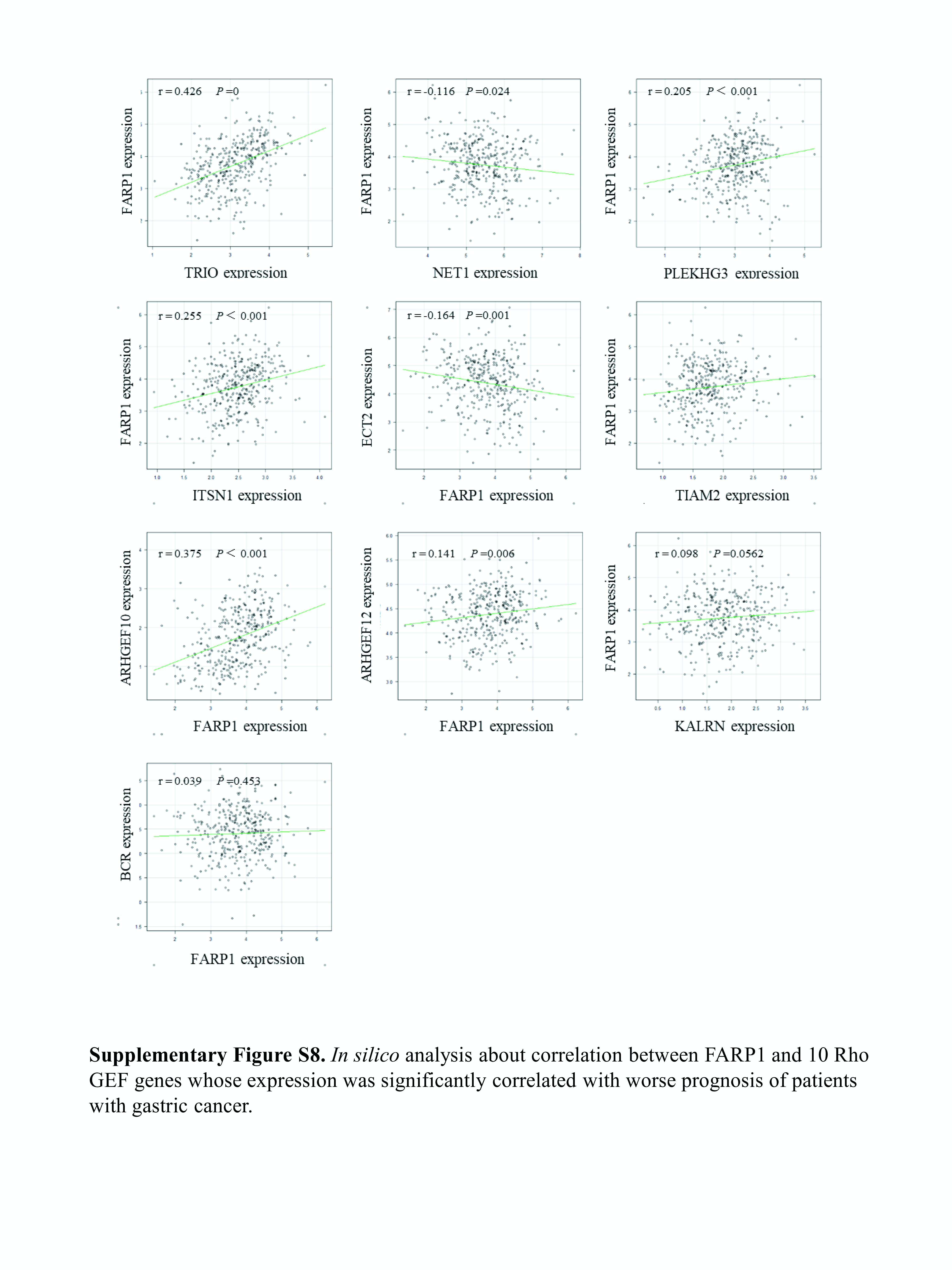

Supplement: Supplementary file 9 — Supplementary Fig.S8 [file 41389_2020_190_MOESM9_ESM.tif]
